# Effect of Various Edge Configurations on the Accuracy of the Modelling Shape of Shell Structures Using Spline Functions

**DOI:** 10.3390/s22197202

**Published:** 2022-09-22

**Authors:** Grzegorz Lenda, Urszula Marmol

**Affiliations:** Faculty of Mining Surveying and Environmental Engineering, AGH University of Science and Technology, Al. Mickiewicza 30, 30-059 Kraków, Poland

**Keywords:** NURBS, splines, modelling, edges, shell structures, laser scanning

## Abstract

Spline functions are a useful tool for modelling the shape of shell structures. They have curvature continuity that allows good approximation accuracy for various objects, including hyperboloid cooling towers, spherical domes, paraboloid bowls of radio telescopes, or many other types of smooth free surfaces. Spline models can be used to determine the displacement of structures based on point clouds from laser scanning or photogrammetry. The curvature continuity of splines may, however, cause local distortions in models that have edges. Edges may appear in point clouds where surface patches are joined, on surfaces equipped with additional technical infrastructure or with cracks and shifts in the structure. Taking the properties of spline functions into account, several characteristic types of edge configurations can be distinguished, which may, to a different extent, affect the values of modelling errors. The research conducted below was aimed at identifying such configurations based on theoretical considerations and then assessing their effect on the accuracy of modelling shell structures measured by laser scanning. It turned out to be possible to distinguish between edge configurations, based on the deviation values.

## 1. Introduction

Spline functions are a useful tool for modelling the shape of shell structures. They have curvature continuity that allows for good approximation accuracy for various curvilinear objects, including hyperboloid cooling towers, spherical domes, paraboloid bowls of radio telescopes, or many other types of smooth free surfaces. Curvature continuity, however, can lead to local distortions of models consisting of patches separated by edges or equipped with additional technical infrastructure. This also applies to surfaces with cracks and shifts in the structure.

The problems associated with the modelling of edges using spline functions are common ones [1,2,3,4,5]. They occur during the modelling of free surfaces in software used in reverse engineering, e.g., Geomagic Design X, Catia, 3D Reshaper, Solidworks with the Scan to 3D module, Rhinoceros with the Rhinoresurf overlay, etc. To eliminate them, various methods are used to create approximation surfaces based on point clouds [6,7,8,9]. Triangular meshes are mainly used [10,11,12], for which the preliminary geometry analysis allows for the design of the patches of spline surfaces so that they do not run across places with large changes in curvature (potential edges). The effectiveness of these methods varies greatly and distortions are rarely avoided.

A single edge is enough to cause distortion, visible as undulations of the smooth shape of an object. However, if there are several edges in close proximity, their mutual influence will accumulate. Undulations are treated as harmful distortions of the model and efforts are made to eliminate or reduce them. The presence of a single edge makes it possible to distribute measurement data along it and model the surface patches separately, which in practice eliminates undulations. However, the close proximity of several edges complicates the separation of point clouds into smaller parts. Modelling such data with spline surfaces usually induces deformations of shell object models that are difficult to remove.

**Figure 1:**
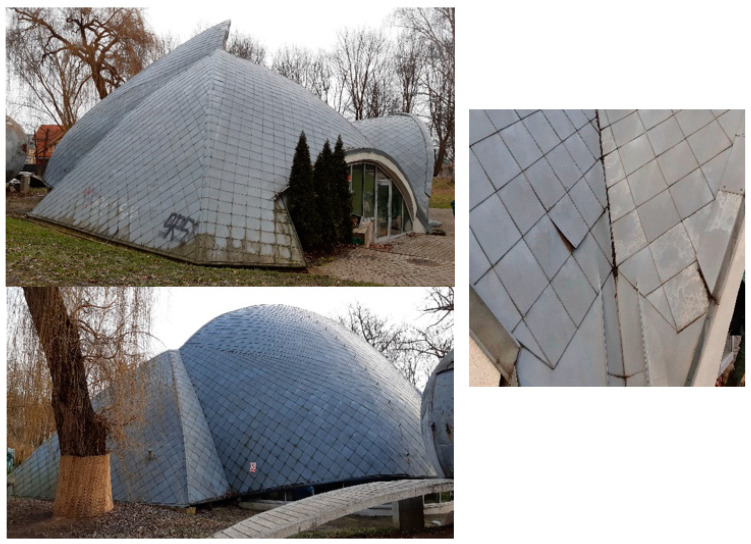
Structure subject to analyses: B. Chromy’s sculpture gallery in Krakow.

As far as the properties of spline functions are concerned, described in detail in the next chapter, it is possible to distinguish several characteristic types of edge configurations which may, to a varying extent, affect the values of modelling errors. No study has so far discussed the effect of the mutual positioning of adjacent edges on the accuracy of modelling with splines. The conducted research was aimed at identifying such configurations based on theoretical considerations and then assessing their effect on the accuracy of modelling shell structures measured by laser scanning. The structure was a shell composed of seven smooth patches separated by sharp edges (Figure 1). It had additional edge elements on the surface, there was also a local crack and displacement of the coating. They formed configurations of several adjacent edges that affected the modelled surfaces in different ways.

The first objective of the research is to evaluate the impact of different edge configurations on the accuracy of modelling with spline surfaces. They will help to know what accuracy can be expected when modelling edges between large surface patches when modelling infrastructure elements covering the surface, and what accuracy can be expected at faults or cracks.

This research is accompanied by a second objective, related to edge recognition of objects in point clouds. Edge recognition is used in remote sensing data processing, e.g., for separating buildings from the airborne lidar point clouds, separating buildings from vegetation, or in general for recognizing components of objects of different scales.

In general, from the point of view of the accuracy of modelled surfaces, undulations occurring along edges are a detrimental phenomenon. However, these same undulations simultaneously indicate the position of the edges, so as a result of analysing the deviation of points from the spline surfaces, the edges of objects can be recognized. Moreover, if the modelling accuracy distinction between the selected edge configurations can be carried out, it is likely that it will be possible not only to recognize edges but also to indicate their configurations, enabling, for example, the separation of infrastructure elements from the object, the detection of cracks, etc. The ability to distinguish edge configurations will thus be a prelude to further research related to the application of spline functions for advanced edge detection.

## 2. Properties of Spline Functions That Determine the Occurrence of Errors When Modelling Edges

Spline functions are the mathematical equivalent of a bar which, bent in terms of its elasticity, retains a shape of continuous curvature (Bernoulli–Euler equation [13,14]):

(1) $$\frac{1}{\rho }=K=\frac{f^{\prime \prime }(x)}{{\left[1+{\left(f^{\prime }(x)\right)}^{2}\right]}^{3/2}}=\frac{M_{z}}{EJ}$$

where: *ρ*—radius of curvature; *K*—curvature; *M_z_*—bending moment; *E*—Young’s modulus; and *J*—moment of inertia.

The spline functions tend to preserve the minimum total curvature, so they have a smooth shape and describe objects with continuous curvatures, such as shell structures, with good accuracy. The convergence of the spline function *S* to the approximated function *f* is expressed by the formula:

(2) $$\varepsilon =\left|f-S\right|\leq \frac{1}{2}\max(h_{i}^{2})M\;\;i=0,..,n$$

where: M=const; $M\geq \left|f^{\prime \prime }(x)\right|$; and $h_{i}=x_{i}-x_{i-1}$.

This estimation highlights that the density of the data has a major impact on the quality of the approximation: when doubling the number of points, you achieve four times less error *ε*. Thus, for modelling, it is reasonable to use high-density point clouds, such as those from laser scanning.

Preserving the curvature continuity requires the continuity of individual segments of the spline function and their two derivatives (C2 class). If the object has kink points (for curves) or sharp edges (for surfaces), it has continuity at these points only of the function (class C0), while the spline function describing it has C2 continuity in the entire domain.

**Figure 2:**
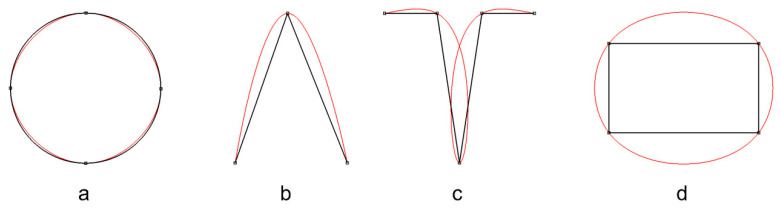
Spline function describing a smooth structure (**a**), with one kink (**b**), and structures with different numbers and locations of kinks (**c**,**d**).

Spline function undulations are formed in such places (Figure 2b) [1,15,16]. Undulations are a detrimental phenomenon from the standpoint of model accuracy, and efforts are being made to remove them. If there are several kinks located close to each other, they can mutually enhance the undulations (Figure 2c,d) and the final effect of the approximation can be unpredictable. Undulations of spline functions can be reduced to some extent by using an appropriate selection of knots [17,18,19,20]; however, in the case of kinks, this is not sufficient.

If a kink is formed, it will affect a total of six function segments, three on each side of it. If there is one kink, the range of the undulations it causes will therefore be local. If there are several kinks and they are close, the range of undulations will extend and their values may increase. In order to analyse the influence of interdependencies between segments, it is necessary to refer to the idea of the construction of spline functions.

Currently, they are most commonly found in the form of NURBS (non-uniform rational B-splines) [1,16,21]:

(3) $$S_{i}(t)=\frac{\sum_{i=0}^{n-m-1}w_{i}d_{i}N_{i}^{m}(t)}{\sum_{i=0}^{n-m-1}w_{i}N_{i}^{m}(t)}\;t_{i}=\{t_{0},\ldots ,t_{n}\}$$

where: $S_{i}(t)$—segment of the NURBS function; $N_{i}^{m}(t)$—*m*-th degree base polynomials; $d_{i}$—control points; $w_{i}$—control point weights; and $t_{i}$—spline knots which are a generalised form of B-spline functions:

(4) $$S_{i}(t)=\sum_{i=0}^{n-m-1}d_{i}N_{i}^{m}(t)\;\;t_{i}=\{t_{0},..,t_{n}\}$$

It is a representation of the spline functions, which is a linear combination of successive base functions $N_{i}^{m}(t)$ with coefficients in the form of control points $d_{i}$. The base functions are determined using the Mansfield-de Boor–Cox recursive formula:

(5) $$N_{i}^{0}(t)=\{\begin{array}{c} 1\;dla\;t\in \left[t_{i},t_{i+1}\right)\\ 0\;dla\;t\notin \left[t_{i},t_{i+1}\right) \end{array}$$

$$N_{i}^{k}(t)=\frac{t-t_{i}}{t_{i+k}-t_{i}}N_{i}^{k-1}(t)+\frac{t_{i+k+1}-t}{t_{i+k+1}-t_{i+1}}N_{i+1}^{k-1}(t)$$

The third-degree polynomials are determined with it, allowing for the formation of a function that preserves curvature continuity, which have the following form:

(6) $$N_{i}^{3}(t)=\frac{{\left(t-t_{i}\right)}^{3}}{\left(t_{i+3}-t_{i})\cdot (t_{i+2}-t_{i})\cdot (t_{i+1}-t_{i}\right)}\;\;\mathrm{dla}\;\;t\in \left[t_{i},t_{i+1}\right)$$

$$N_{i}^{3}(t)=\frac{\left(t-t_{i})\cdot (t_{i+3}-t)\cdot (t-t_{i+1}\right)}{\left(t_{i+3}-t_{i})\cdot (t_{i+3}-t_{i+1})\cdot (t_{i+2}-t_{i+1}\right)}+\frac{(t_{i+4}-t)\cdot {\left(t-t_{i+1}\right)}^{2}}{\left(t_{i+4}-t_{i+1})\cdot (t_{i+3}-t_{i+1})\cdot (t_{i+2}-t_{i+1}\right)}+$$

$$+\frac{(t_{i+2}-t)\cdot {\left(t-t_{i}\right)}^{2}}{\left(t_{i+3}-t_{i})\cdot (t_{i+2}-t_{i})\cdot (t_{i+2}-t_{i+1}\right)}\;\;\mathrm{dla}\;\;t\in \left[t_{i+1},t_{i+2}\right)$$

$$N_{i}^{3}(t)=\frac{(t-t_{i})\cdot {\left(t_{i+3}-t\right)}^{2}}{\left(t_{i+3}-t_{i})\cdot (t_{i+3}-t_{i+1})\cdot (t_{i+3}-t_{i+2}\right)}+\frac{(t-t_{i+2})\cdot {\left(t_{i+4}-t\right)}^{2}}{\left(t_{i+4}-t_{i+1})\cdot (t_{i+4}-t_{i+2})\cdot (t_{i+3}-t_{i+2}\right)}+$$

$$+\frac{\left(t_{i+4}-t)\cdot (t-t_{i+1})\cdot (t_{i+3}-t\right)}{\left(t_{i+4}-t_{i+1})\cdot (t_{i+3}-t_{i+1})\cdot (t_{i+3}-t_{i+2}\right)}\;\;\mathrm{dla}\;\;t\in \left[t_{i+2},t_{i+3}\right)$$

$$N_{i}^{3}(t)=\frac{{\left(t_{i+4}-t\right)}^{3}}{\left(t_{i+4}-t_{i+1})\cdot (t_{i+4}-t_{i+2})\cdot (t_{i+4}-t_{i+3}\right)}\;\;\mathrm{dla}\;\;t\in \left[t_{i+3},t_{i+4}\right)$$

$$N_{i}^{3}(t)=0\;\;\;\mathrm{dla}\;\;t\notin \left[t_{i},t_{i+4}\right)$$

**Figure 3:**
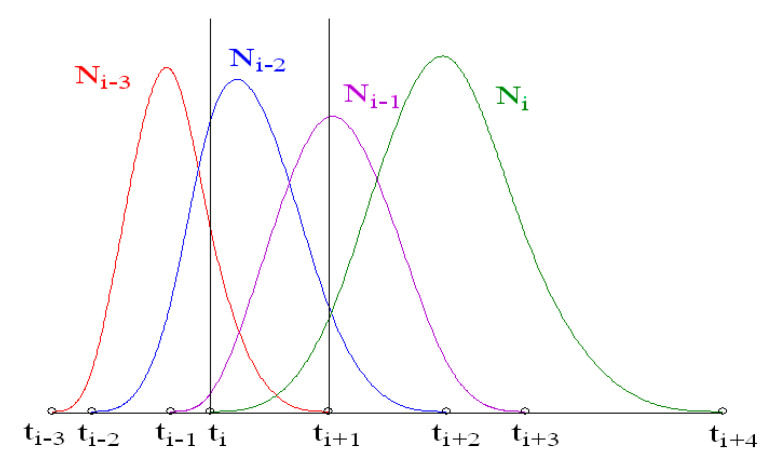
Third-degree base polynomials $N_{i}^{3}(t)$, each defined on four consecutive intervals. In the interval $\left[t_{i},t_{i+1}\right)$, there are four base polynomials involved in determining a single segment of the B-spline.

Each third-degree base polynomial $N_{i}^{3}(t)$ is constructed based on four consecutive intervals $\left[t_{i},t_{i+1}\right)$, outside of which it takes the value of zero (Figure 3). Four base polynomials take part in determining the cubic B-spline function on the interval $\left[t_{i},t_{i+1}\right)$ (Figure 3):

Thus, each segment of a B-spline function can be written as:

(7) $$S_{i-3}(t)=d_{i-3}N_{i-3}^{3}(t)+d_{i-2}N_{i-2}^{3}(t)+d_{i-1}N_{i-1}^{3}(t)+d_{i}N_{i}^{3}(t)$$

**Figure 4:**
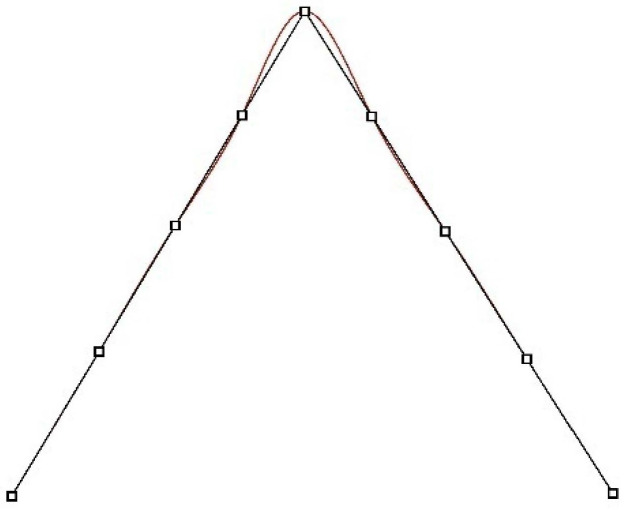
Influence of undulations formed in the vicinity of a kink on the segments of a spline.

It is easy to observe that the base polynomials forming the function segment in the interval $\left[t_{i},t_{i+1}\right)$ also occur in the three intervals before it and the three intervals after it (Figure 3). Therefore, they affect six adjacent segments of the B-spline. If there is a kink in the shape of the object described by the spline, it will cause undulations that will occur in the corresponding number of sections (Figure 4). The greatest undulations are visible in the first section at the kink, significantly smaller in the next one, they are barely noticeable in the third one, and in the fourth, they disappear. If several kinks are close to each other, their mutual influence is difficult to predict. It depends on their number, the number of separate segments, the selection of the knots of the spline function, and the shape of the function in the immediate vicinity.

The number of segments is strictly related to the number of points the B-spline passes through, but only in the case of interpolation when each segment of the function passes through pairs of consecutive points. When spline models are based on laser scanning data, approximation B-spline functions are used (6) [18,22,23], which reduce the measurement noise and decrease the number of function segments in relation to the number of measured points, which greatly increases computation speed.

(8) $${\sum_{j=0}^{r}\left[p_{j}-\sum_{i=0}^{n-m-1}d_{i}N_{i}^{m}(t)\right]}^{2}\to \min$$

where: *n − m*—number of control points of a spline and *r* + 1—number of data points $p_{j}$.

This process, however, extends the effect of the undulations occurring around the kinks. For example, two adjacent kinks may be 30 points apart from the laser scanning measurement. In the case of interpolation, the mutual influence of the undulations accompanying these kinks will not occur. However, during the approximation, one segment of the B-spline can be formed by any number of points. If there are 10 of them per segment on average, then two kinks will be distant by only three function segments and the mutual effect of undulations will expand.

The considerations so far were carried out for 2D curves. When modelling the surface, however, approximate B-spline surfaces are used, created using the *lofting* technique (7) [6,9,24,25]:

(9) $${\sum_{j=0}^{r}\left[p_{j}-\sum_{i=0}^{n-m-1}\sum_{j=0}^{k-m-1}d_{i,j}\cdot N_{i}^{m}(t)\cdot N_{j}^{m}(u)\right]}^{2}\to \min\;\;t_{i}=\{t_{0},..,t_{n}\}\;\;\;u_{i}=\{u_{0},..,u_{r}\}$$

**Figure 5:**
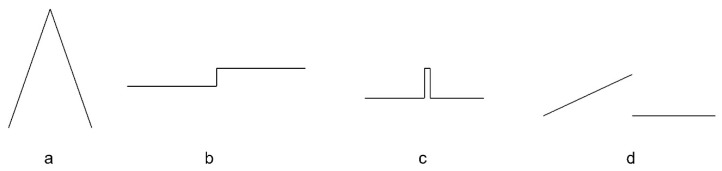
Configurations of surface edges (cross-sectional view—kink points correspond to the sharp edges): (**a**) single edge separating patches; (**b**) closely adjacent two edges; (**c**) closely adjacent four edges; (**d**) two edges with a gap (unspecified connection)—cracks and displacements.

As far as surfaces are concerned, the analysed kink takes the form of a sharp edge of two adjacent patches. Edges can come in several configurations which, according to theoretical considerations, may cause undulations to overlap and ultimately increase their mutual effect. These configurations are illustrated in Figure 5 (cross-sectional view).

The first configuration (a) is the simplest case of a single edge that mostly separates two surface patches that are not connected with continuous curvature.

The second configuration (b) is a surface step which may be associated with installation elements or be the edge of the structure (flange). The proximity of the two edges may increase the mutual effect of the undulations.

The third configuration (c) is an elongated narrow element which may be a stiffening element or an installation element. The proximity of the four edges may further increase the mutual effect of undulations. In the case of very narrow elements, this configuration can be treated as a three-edge configuration because no segment of the approximation function will fit between the upper points.

The fourth configuration (d) represents a crack and displacement of the surface. The shape of the surface is undetermined between the edges as there is no connection, as a result of a gap in the set of points. As in the case of (b); there are two edges.

## 3. Characteristics of the Research Object and Preparation of Data for Accuracy Analyses

**Figure 6:**
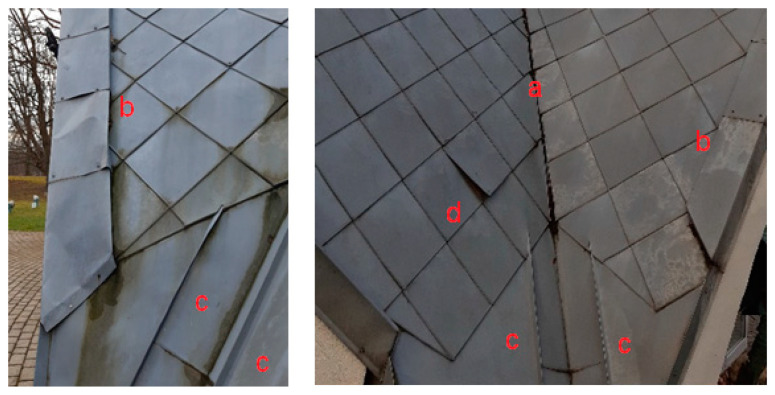
Configurations of edges of the analysed shell: (a) single edge separating patches; (b) closely adjacent two edges; (c) closely adjacent four edges; (d) two edges with an undetermined connection—crack and displacement.

The analysed structure has all the mentioned edge configurations. The creation of the spline model will allow for assessing where the undulations will occur and what their values will be. Single edges separating the surface patches are illustrated in Figure 1 and Figure 6 (configuration a). Figure 6 presents a flange along the front of the structure, constituting a configuration of the two close edges (b). In the vicinity of the concave edge (a), longitudinal metal plates transverse to the surface are also visible, constituting the configuration of the four close edges (c). The right figure also illustrates a plate detached from the shell, corresponding to the configuration of the two edges without the connection (configuration d).

**Figure 7:**
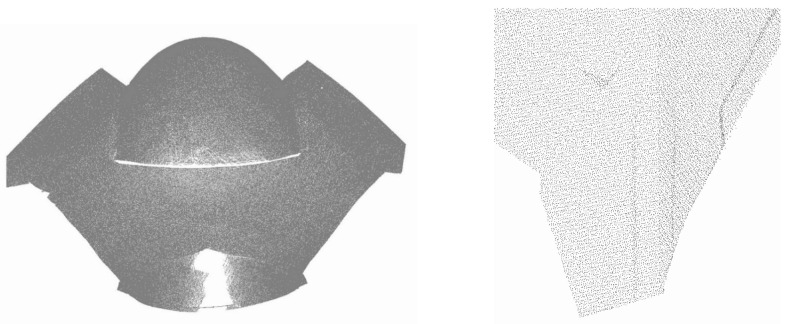
Point cloud of the structure and its fragment containing edges presented in Figure 6.

The structure was measured with a Leica ScanStation C10 laser scanner with a point accuracy of 6 mm and 2 mm precision. The average point cloud resolution was 5 mm. The average registration error of point clouds equals 3 mm. The edges from the right in Figure 6 are visible on the cloud fragment (Figure 7).

To create the model, approximated B-spline surfaces (Formula (9)) were used, created using the lofting technique. An iterative process was used, for which the tolerance of fitting the surface into a set of points was set. An appropriate selection of nodes was also used, affecting the shape of the function. The spline surface was constructed in the RhinoResurf programme for creating an approximation of B-spline surfaces based on point clouds. It is an overlay to the Rhinoceros modelling software using spline functions that was used for further analysis. First, a dense mesh was constructed for the point cloud, which is the first modelling step. Based on the geometry read from the mesh, allowing, e.g., for the automatic determination of knots for the approximation patches, spline surfaces were iteratively determined in the next step. Iterations were performed for the theoretical tolerance of fitting the surface to the point cloud set at the value of 0.1 mm. In practice, however, such tolerance cannot be achieved due to the measuring noise of the laser scanner.

A selection of nodes (function arguments) proportional to the distance between points was used [18], suitable for shell objects.

The analyses performed were affected by several disturbing factors. The first one was related to the accuracy of the point measurement for the scanner used, which was 6 mm. However, the surface modelling error, which was more closely associated with the fitting of the patches to the noisy data, was specified by the manufacturer at 2 mm. Taking into account the metal coating of the surface, which is conducive to errors when measuring at acute angles, and the occurrence of such angles, especially for the upper zones of the structure, higher measurement noise could be expected in some places.

Another disturbance was the measurement resolution. It was set at 5 mm, but in the upper parts of the structure, measured at acute angles, it could decrease. The upper parts of the single edges separating the individual patches and the upper part of the front flange were located in such places. Due to the lower position of the scanner positions in relation to the front part of the structure shell, it was not possible to measure it, as shown in Figure 7.

The last disturbing factor was related to the initial mesh modelling. The mesh of maximum density was used. When creating the mesh, however, triangles could be generated transversely to the edge, which affected the distortions of the modelled edge. Decreasing the point cloud density increased these distortions.

All these factors will occur with particular intensity in the upper parts of the surface, due to the measurements performed at acute angles, greater rarefaction of the data and the accompanying rarefaction of the mesh. However, in the upper parts, there are only the edges separating the patches, so it will be possible to compare the accuracy of their lower and upper parts. The influence of these factors on modelling the remaining edge configurations at the lower part of the surface will be smaller. First of all, it will be mutually comparable in these places, allowing for observing differences between different edge configurations, modelled using spline functions.

The analyse of the accuracy of fitting the surface to the point cloud was carried out using deviation diagrams, grouping them into five intervals with the values: [0, 5), [5, 10), [10, 15), [15, 20) and [20, 50] mm. The first interval with deviations up to 5 mm could be interpreted as correctly formed surface fragments falling within the measurement noise range, including elements with less favourable scanning geometry.

For each edge, the values of mean and maximum deviations as well as the percentage share of deviations in the intervals were determined. It should be emphasised that these were proximate indicators. It would be impossible to distinguish individual patches on each side of the edge from the created surfaces, especially since the edges could be uneven. Therefore, a decision was made to choose the vicinity of the edge in which deviations of increasing values resulting from undulations occurred. For places with low measurement noise, deviations related to undulations may be noticeable even at values slightly exceeding 2 mm. Thus, the cut-off threshold for deviations around the edges was set at 3 mm. This is not a strict procedure for distinguishing the edges, but it allowed us to unambiguously classify the undulations caused by different edge configurations.

**Figure 8:**
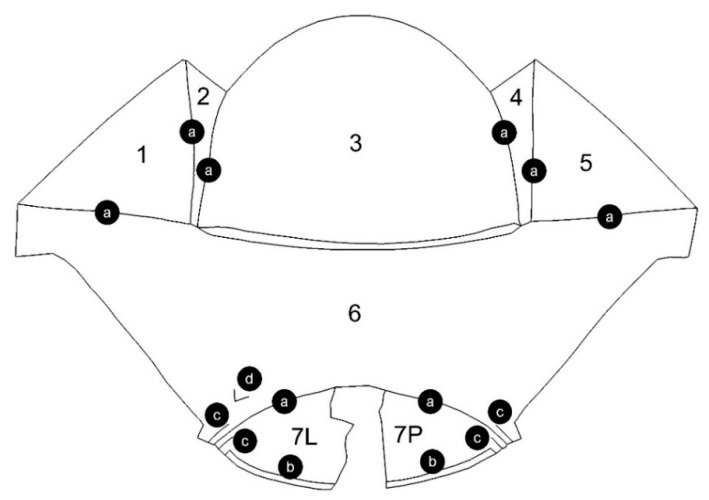
Numbering of surface patches: 1–7 and denotation of edge configurations: (a) one-edge; (b) two-edge; (c) four-edge; (d) two-edge with a gap.

Figure 8 illustrates the numbering of the surface patches and the denotation of the edge configurations: (a) one-edge, (b) two-edge, (c) four-edge, and (d) two-edge with a gap. Patch no. 7 could not be fully modelled due to the lack of observations in the upper part of the point cloud (Figure 7).

## 4. Analysis of a Spline Model of a Shell Structure Containing Edges in Various Configurations

**Figure 9:**
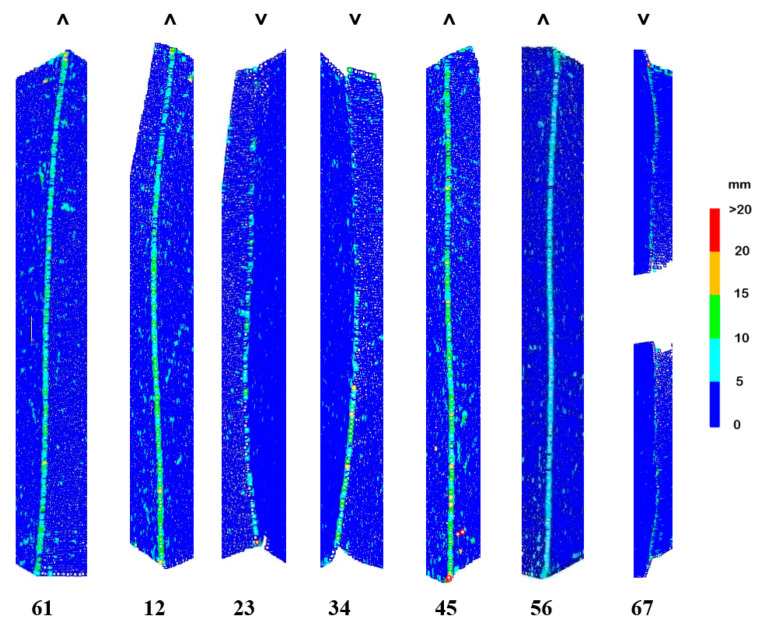
Point cloud deviations from the surface along single edges separating main patches of the structure. The symbols ˅ and ˄ denote concave and convex edges.

First, the shape of spline surfaces along the single edges separating the patches of the shell structure was checked (Figure 9). The numerical analyses are contained in Table 1.

**Figure 10:**
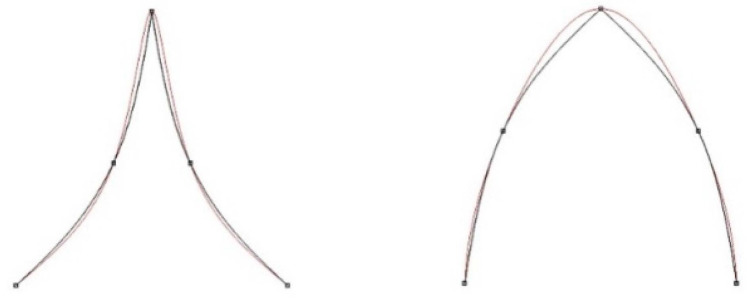
Undulations of the spline in the vicinity of kink point of curves making concave and convex connections.

Deviations occurred along each single edge. Interestingly, a better approximation was obtained for the concave edges: 23 and 34 than for the convex edges: 12, 45, 61 and 67. The data for the concave edge 67 were not representative due to tree leaves lying there, which increased modelling errors. More than 90% of the deviations for single edges did not exceed the value of 15 mm. Mean deviations for convex edges (8.5 mm) were about 1.5 times greater than for concave edges (5.5 mm). The experiment on theoretical data confirmed the observation that the undulations in the area of the kink for the curves forming the concave connections were smaller than for the convex connection (Figure 10).

In the upper parts of the edges, exposed to the aforementioned disturbing factors, no increase in the value of deviations was observed. Moreover, the concave edges 23 and 34 were smaller than at the bottom. Thus, it could be observed that the disturbing factors caused deviations in the smallest range of [0, 5) mm and did not affect the edge analysis.

**Figure 11:**
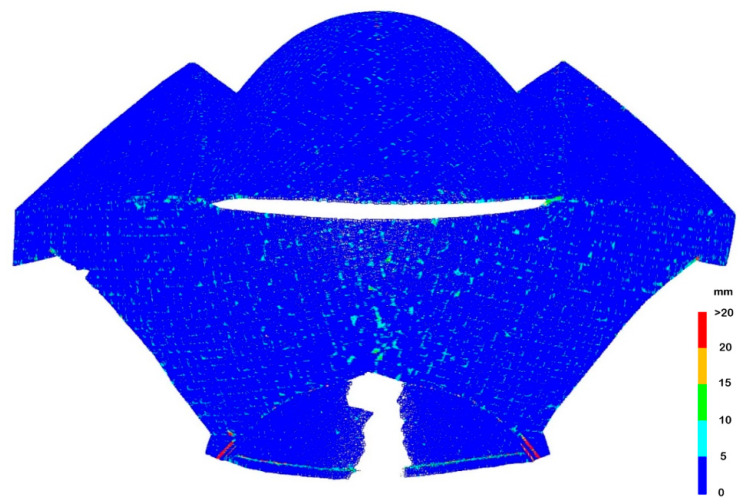
Deviations of point cloud from spline model for which patches separated by single edges were modelled separately.

The presence of single sharp edges between the surface patches allowed them to be easily separated, and then the patches could be created based on independent point clouds. Thus, it was possible to avoid the local dependence of the segments of the splines at the edge. This method was used to create the model of the entire structure, which was analysed further. The deviations of the point cloud from the model are illustrated in Figure 11.

**Figure 12:**
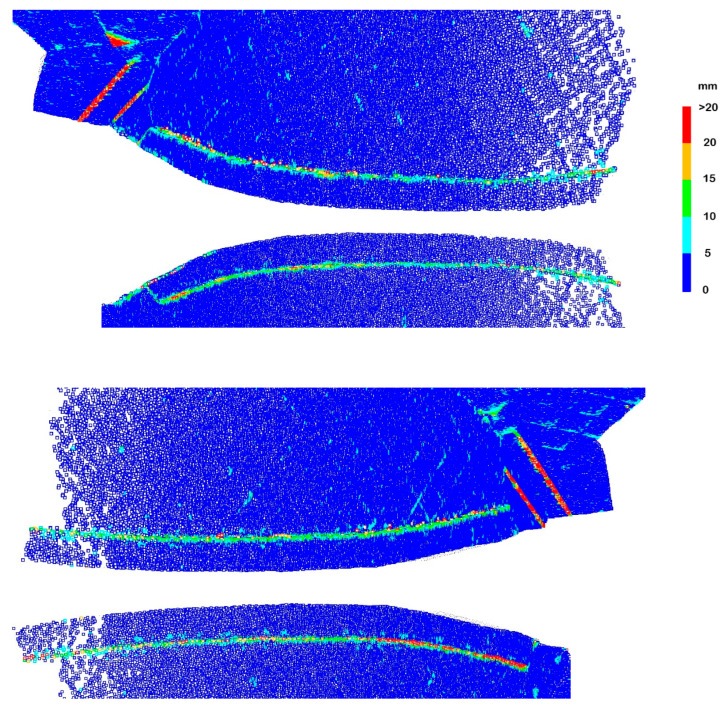
Deviations for edge configurations in Figure 8: two-edge, four-edge, two-edge with a gap. Views of the left and right parts of surfaces 6 and 7, flange of surface 7 visible from outside and inside.

The deviations that occurred along the single edges were eliminated because there was no local dependence of the segments of the spline function on both sides of the edge. Generally, high accuracy of fitting the whole model was achieved, the mean deviation was 1.6 mm. However, in the places marked in Figure 8 as multi-edge configurations, deviations of large values occurred. They are presented in enlargement in Figure 12. The deviations of the flange along patch 7 obscured each other in the external and internal views, therefore both views are presented.

The two-edge configuration was along a flange located in front of patch 7. There was a vertical step of about 4 cm between the patch and the flange. The edges were so close to each other that the created approximation patches directly influenced each other. This resulted in an increase in the value of deviations compared to single-edge configurations to an average of 12.5 m. About 15% of the deviations fell within the range of [15, 20) mm, and about 8% above 20 mm. The maximum deviations also increased from 10–17 mm to 25–28 mm. Clusters of larger deviations appeared at the bottom of the edge.

Undulations accompanying a two-edge configuration are more difficult to remove than single-edge ones. With single edges, it is enough to separate the clouds along the edges. With two-edge ones, there is a narrow strip of points between the main patches which, in the case of curvilinear structures, is difficult to remove manually. Its possible residues will cause undulations associated with single-edge configurations.

The four-edge configurations extended along narrow sheet metal strips located near the lower joints between patches 6 and 7. They were small, several millimetres thick, and approximately 3 cm high. These edges were therefore very closely adjacent to each other, which entailed the mutual influence of the undulations. As a result, the obtained deviation values were greater than in the case of single-edge and two-edge configurations. The mean values of the deviations increased significantly and reached approximately 19 mm. The maximum deviations remained at a level similar to the two-edge configuration and they were 26–30 mm. More than half of the deviations reached values above 20 mm, and in total, approximately 75–90% exceeded 15 mm.

Undulations in the four-edge configuration can be eliminated by removing long two-dimensional elements from the surface. For curved patches, this is relatively easy on the convex side, but difficult on the concave side.

**Figure 13:**
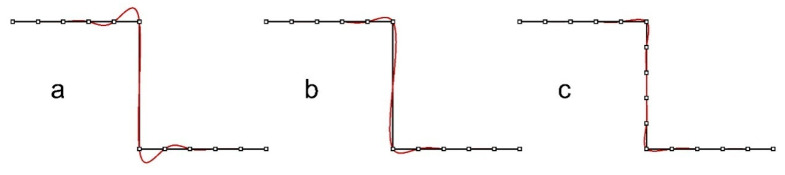
Two-edge configurations: with a gap (knot vector: (**a**)—uniform, (**b**)—centripetal), (**c**)—ordinary two-edge configuration with no gap in points.

A two-edge configuration with a gap occurred for a cracked and protruding plate of sheet metal coating the shell. It was located in the lower left part of patch 6. The tile protruded from the surface by about 4 cm, which meant that there were no points between the edges. It could be observed that groups of deviations with larger values occurred more frequently than for the normal two-edge configuration. The lack of points between the edges placed the edges in the immediate vicinity, enhancing the undulation effect. An additional factor generating undulations is the selection of knots of spline surfaces, which in the case of gaps in point clouds is sometimes difficult. Figure 13 illustrates a two-edge shape with a point gap. In the case of (a), the uniform knot vector was used [15,18], which gave large distortions in the vicinity of the gap. In the case of (b), the centripetal knot vector was used [15,18], which resulted in a reduction of distortions and a slightly different distribution. The case of (c) presents a structure without a gap in the point cloud for which distortions are the smallest. As it is impossible to fill in the lack of points at the crack, two-edge configurations with a gap generate more undulations than regular two-edge configurations.

The mean deviation for this configuration was 18 mm, and the maximum was 41 mm. The deviation intervals were arranged in layers increasing towards the crack. The largest group of deviations was the most numerous (40%).

## 5. Conclusions

The conducted theoretical considerations allowed for the selection of several configurations of surface edges, which could affect the accuracy of modelling the patches using spline functions in various ways. The studies verifying the assumptions made for the real structure confirmed the reasons for dividing the edges into four configurations: one-edge, two-edge, four-edge and two-edge with a gap. They differed in the value of induced distortions and the possibility of their potential elimination. Average/maximum distortions reached on average the following values: 7/14 mm (one-edge), 12/26 mm (two-edge), 19/27 mm (four-edge) and 18/41 mm (two-edge with a gap). Considering the average accuracy of 1.6 mm obtained when fitting the surface into the point cloud, these are significant deviations. As a result of the research, it is possible to assess what accuracy should be expected when modelling edges between large patches of the surface, what accuracy should be expected when modelling infrastructure elements covering the surface, and what accuracy should be expected at faults or cracks. This was the first objective of the research.

Precise modelling of the surface is related to the effective elimination of undulations originating from the described configurations. Eliminating one-edge configurations is as simple as separating point clouds along the edges. Such a procedure was carried out in the second part of the modelling. For two-edge configurations, distinguishing/removing a narrow strip of points between the patches is difficult to perform manually. The possibility of easily removing four-edge configurations depends on their position on the convex or concave side of the patch. The two-edge configuration with a gap contains a gap in the observations that increases the undulations. It is not possible to remove it, but only to try to fill it with points, which will, however, subjectively change the shape of the model.

As it turned out to be possible to distinguish between edge configurations, the observed properties can be seen as a prelude to edge detection in laser scanning point clouds, which was the second objective of the study. With the analysis of point deviations from the surface, all locations of the object’s internal edges were correctly identified. However, it would have been useful to better distinguish between the deviation values for different edge configurations, which would have made it easier to assess whether they originate from cracks, faults, or features on the object’s surface. The authors estimate that this can be achieved, for example, by using different algorithms for node selection, weighting NURBS functions, or manipulating surface fitting tolerance. Since all studies related to the modelling of spline surfaces tend to minimize surface undulations and edge group detection requires the opposite process, i.e., a controlled increase in undulations, it is difficult to assess for the time being which of the aforementioned methods will yield the best results, therefore this requires further research.

The computational cost of the edge recognition method using spline surfaces is equal to the cost of creating the surface and calculating the deviation of points from the surface. Determination of the spline surface for a building object measured by laser scanning (clouds of several hundred thousand to several million points) on modern computers takes an average of several to tens of minutes. Determination of the spline surface is a sequential process, loading one processor core, so multi-threaded processors do not speed up calculations. The analyzed surfaces were created in RhinoResurf at a time of 4 min.

## Figures and Tables

**Table 1:** Mean, maximum and interval deviations for each edge configuration.

Edge	Path	Deviation [mm]		Percentages of Deviations [mm]			
Configuration	Position	Mean	Max	>20	[15, 20)	[10, 15)	[3, 10)
**1-edge**	23 concave	5	10	0	2	25	73
	34 concave	6	13	0	5	19	75
	67 concave	7	19	6	13	28	54
	61 convex	8	16	0	4	40	57
	12 convex	9	15	0	1	37	62
	45 convex	9	17	0	5	39	55
	56 convex	8	15	0	3	38	59
**2-edge**	7 left	12	25	6	16	41	36
	7 right	13	28	10	15	37	38
**4-edge**	6 left	20	30	54	22	13	11
	7 left	17	27	45	29	19	7
	6 right	19	27	58	25	8	9
	7 right	20	26	51	40	7	2
**2-edge with gap**	6 left	18	41	40	22	24	14

## Data Availability

Not applicable.
